# Determination of temporal reproducibility and variability of cancer biomarkers in serum and EDTA plasma samples using a proximity extension assay

**DOI:** 10.1186/s12014-022-09380-y

**Published:** 2022-11-15

**Authors:** Troels D. Christensen, Emil Maag, Kasper Madsen, Sidsel Christy Lindgaard, Dorte L. Nielsen, Julia S. Johansen

**Affiliations:** 1 Department of Oncology, Copenhagen University Hospital - Herlev and Gentofte, Borgmester Ib Juuls Vej 1, 2730 Herlev, Denmark; 2 BioXpedia, Aarhus, Denmark; 3 Department of Clinical Medicine, Faculty of Health and Medical Sciences, University of Copenhagen, Copenhagen, Denmark; 4 Department of Medicine, Copenhagen University Hospital - Herlev and Gentofte, Herlev, Denmark

**Keywords:** Olink immuno-oncology, Proximity extension assay, Multiplex Immunoassays, Proteins, Tumor markers, Serum-plasma variability

## Abstract

**Background:**

Proximity extension assay (PEA) is a novel antibody-based proteomic technology. Sparse data have been published concerning the matrix effect of serum vs. ethylenediamine tetraacetic acid (EDTA) plasma and the reproducibility of results obtained using PEA technology.

**Methods:**

We analyzed samples with the PEA-based 92-plex Olink® immuno-oncology (I-O) assay. To estimate the matrix effect, we analyzed paired serum and EDTA plasma samples from 12 patients with biliary tract cancer. To evaluate the reproducibility, we used data from 7 studies, where 6–8 serum samples from patients with pancreatic cancer were used as bridging samples on 3 versions of the panel over a 2.5-years period.

**Results:**

For the study of serum vs. plasma, 80 proteins were evaluable. The mean serum to EDTA plasma ratio ranged from 0.41–3.01. For 36 proteins, the serum and plasma values were not comparable due to high variability of the ratio, poor correlation, or possible concentration effect. For the bridging samples, the mean intra-study inter-assay coefficient of variation (CV) ranged from 11.3% to 26.1%. The mean inter-study CV was 42.0% before normalization and 26.2% after normalization. Inter-study results were well correlated (*r* ≥ 0.93), especially for studies using the same version of the panel (*r* ≥ 0.99).

**Conclusion:**

For 44 of 92 proteins included in the Olink® I-O panel, the variation between results obtained using serum and EDTA plasma was constant and results were well correlated. Furthermore, samples could be stored for several years and used on different versions of the same PEA panel without it effecting results.

**Supplementary Information:**

The online version contains supplementary material available at 10.1186/s12014-022-09380-y.

## Introduction

There is an increasing interest in developing panels of cancer biomarkers for diagnostic, prognostic, and predictive applications [1, 2]. Attention has shifted from single biomarker measurements to using multiple biomarkers in combinations to achieve better discriminatory capabilities [3–5]. This shift has been aided by the development of new technologies. A novel proteomic technology for biomarker research is the antibody-based proximity extension assay (PEA). This method enables simultaneous measurement of many proteins (from 92 to 384) at low abundance [6]. The PEA technology is based on protein detection by pairs of antibodies linked to DNA oligonucleotides (probes). When a pair of antibodies binds to a target protein, the probes are brought into proximity, hybridized, and extended by a proximity-dependent DNA polymerization event, followed by quantification using real-time polymerase chain reaction (qPCR) [6]. Today more than 500 publications have reported use of this method in biomarker studies, including more than 90 cancer biomarker publications [7–12]. One of the most widely used PEA panels in cancer research is the 92-protein immuno-oncology (I-O) panel from Olink® proteomics (Uppsala, Sweden) [10–12].

Biomarker studies are often based on sparse and valuable clinical samples from biobanks used in multiple studies. Furthermore, it may not always be possible to obtain the same blood sample matrix from patients in different studies. Two of the most used blood-derived products for proteomics are serum and ethylenediamine tetraacetic acid (EDTA) plasma. Although both matrices can be analyzed using PEA technology, results obtained with the two sample types cannot be directly compared. The only published data quantifying the variation between protein levels in serum and EDTA plasma samples using the Olink® I-O panel are based on matched samples from only 4 healthy subjects [13].

Another methodological problem is the comparison of biomarker results obtained in analyses performed using different array plates over several points in time. When using PEA technology, the proteins are measured as normalized protein expression (NPX) values which are relative quantifications [6]. Therefore, it is not possible to compare NPX values obtained using different plates without normalization using bridging samples. The same bridging samples might be used for several years, enabling researchers to compare the results obtained in different studies.

In this study, we used the PEA-based I-O panel from Olink® and evaluated the variation and correlation between protein results in serum and EDTA plasma samples in patients with cancer. We also assessed the temporal reproducibility and the effect of storage using bridging samples analyzed in several studies over 3 years using different versions of the I-O panel.

## Materials and methods

### Study cohort and sample preparation for sub-study of differences between protein levels in serum and plasma samples

We analyzed matched serum and EDTA plasma samples from 12 patients with advanced biliary tract cancer included between October 2020 and May 2021 in the CHOCA study—an ongoing prospective observational open-cohort biomarker study of patients with biliary tract cancer. The study was approved by the Regional Danish Ethics Committee (approval number: H-3–2014-055). The samples for serum and plasma were drawn simultaneously prior to first line palliative chemotherapy. The blood samples were drawn into 8-ml serum tubes (Vacuette® Tube 8 ml CAT Serum Separator Clot Activator) and 9-ml EDTA tubes (Vacuette® Tube 9 ml K2EDTA). According to the standard operating procedure (SOP) of the CHOCA biomarker protocol, the tubes were stored at room temperature for 30 to 120 min before they were centrifuged at 2300 g at 4 °C for 10 min. Serum and EDTA plasma samples were then aliquoted into Greiner tubes (Cryo.s™ Freezing tubes, 2 ml, GR-121280, Greiner Bio-One GmbH) and subsequently stored at − 80 °C until analysis. For the analysis we only selected samples without visible hemolysis. The Regional Danish Ethics Committee had approved the study (H-3–2014-055).

### Study cohort and sample preparation for sub-study of temporal reproducibility and effect of storage

The bridging serum samples were collected from 8 patients with advanced pancreatic cancer included between March 2017 and July 2018 in the “BIOmarkers in Patients with Pancreatic Cancer (BIOPAC)” study—an ongoing prospective observational open-cohort biomarker study (Regional Danish Ethics Committee approval number: KA-20060113; ClinicalTrials.gov ID: NCT03311776; www.herlevhospital.dk/BIOPAC/). All samples were drawn prior to first line palliative chemotherapy. The 8 bridging samples were analyzed in 7 studies at 7 different time points between November 2018 and June 2021. A description of each study is available in Table 1.

**Table 1:** Description of original studies from where data was used

Study	Description of the study	Date of analyses	Version of Olink I-O panel	N plates	N bridging samples included on each plate
BX0144	Protein biomarkers for PC – part of the BIOPAC study	22-Nov-2018 to 06-May-2019	v. 3101	19^a^	8
BX0182	Protein biomarkers for cancer diagnosis	01-Oct-2019 to 08-Oct-2019	v. 3111	9	8
BX0188	Protein biomarkers for malignant melanoma	10-Dec-2019	v. 3111	3	7
BX0202	Protein biomarkers for immunotherapy efficacy in PC	24-Mar-2020	v. 3111	2	6
BX0214	Protein biomarkers for colorectal cancer	13-Mar-2020	v. 3111	1	8
BX0223	Protein biomarkers for BTC – discovery – CHOCA study	16-Nov-2020	v. 3111	5	8
BX0263	Protein biomarkers for BTC – validation – CHOCA study	08-Jun-2021	v. 3112	1	8

^a^14 out of 19 plates included all 8 samples. On the last 5 plates, the 8 samples were distributed evenly, and for the analysis they were regarded as analyzed on the same plate

*I-O* immuno-oncology, *v* version, *N* number, *PC* pancreatic cancer, *BTC* biliary tract cancer, *CRC* colorectal cancer

Serum samples were collected and processed according to the SOP of the BIOPAC protocol, identical to the processing in the CHOCA protocol (described above). To prepare the bridging samples, the frozen serum samples were thawed at room temperature and centrifuged at 3116 g at 4 °C for 10 min. Each serum was then aliquoted into 11 different Greiner tubes in aliquots of 200 ul and subsequently stored at − 80 °C until analysis of the Olink I-O assay at BioXpedia (Aarhus, Denmark).

### Olink I-O assay

We used the 92-plex Olink I-O assay (Olink® Proteomics, Uppsala, Sweden). Data regarding precision, detection limit, measuring range and analytical specificity have been published by the manufacturer [13].

The analyses were performed at BioXpedia A/S, Aarhus, Denmark (www.bioxpedia.com). For the analyses, serum and EDTA plasma samples were thawed, mixed using a vortex mixer, and centrifuged at 400 g for 1 min. Then 1 μL of serum or EDTA plasma was manually transferred and mixed with the antibody-probe mix and analyzed according to the manufacturer’s instructions. Matched samples were analyzed side by side on the plate. All samples for the serum-plasma variability sub-study were analyzed on the same plate. As recommended by Olink, we also included two sample controls (pooled serumsamples from the study), two negative controls, and two inter-plate control (synthetic samples) on each plate. According to the manufacturer’s recommendations, all samples were normalized for any plate effects using the built-in inter-plate controls. Results were reported as NPX on a log 2 scale.

All samples for the serum-plasma variability sub-study were analyzed on the same plate using Olink Target 96 I-O assay v. 3112.

To investigate temporal reproducibility, we used results from six different studies where the same bridging samples (up to 8) had been analyzed. In four studies (BX0182, BX0214, BX0223), all 8 bridging samples were included on all plates. In BX0144, all 8 bridging samples were included on each of 14 of 19 plates. On the last five plates, the eight samples were distributed randomly, and for the analysis they were regarded as analyzed on the same plate. In BX0263, the eight bridging samples were only included on one plate. In BX188 and BX0202, only seven and six bridging samples were included and analyzed on each plate. During the study period, the manufacturer changed the versions and names of their I-O panel. In the first study, BX0144, we used the older version (v.) 3101 (Proseek® Multiplex Immuno-Oncology). In the five subsequent studies (BX0182, BX0188, BX0202, BX0214, BX0223), we used v. 3111 (Olink Target 96 I-O), and in the newest study (BX0263) we used v. 3112. All versions covered 92 proteins (protein list available in Table 2). However, four proteins were changed between v. 3101 and v. 3111; interleukin (IL) 21 (IL21), IL35, interferon (IFN) beta, vascular endothelial growth factor C (VEGFC) were only used in the older version. These were replaced by the following four in the newer version: lymphocyte activation gene 3 protein (LAG3), IL15, mucin-16 (MUC-16), and killer cell immunoglobulin-like receptor 3DL1 (KIR3DL1). Furthermore, the assays had been changed for tumor necrosis factor (TNF) and IFN-gamma between the older and newer versions. No changes were made between v. 3111 and v. 3112. Lastly, when comparing results between studies, proteins were removed if they did not pass quality control in at least one study. When comparing all six studies, five proteins were removed for this reason (IL1 alpha, IL2, IL13, IL33, and arginase-1 [ARG1]). Therefore, only 81 proteins were in common in all six studies.

**Table 2:** List of proteins included in the study

	Protein name	UniProt ID	Olink I-O v. 3101	Olink I-O v. 3111 and 3112	Plasma/serum ratio^a^	Temporal stability^b^
ADA	Adenosine deaminase (ADA)	P00813	+	+	X	X
ADGRG1	Adhesion G-protein coupled receptor G1 (ADGRG1)	Q9Y653	+	+	X	X
ANGPT1	Angiopoietin-1 (ANGPT1)	Q15389	+	+	X	X
ANGPT2	Angiopoietin-2 (ANGPT2)	O15123	+	+	X	X
ARG1^c^	Arginase-1 (ARG1)	P05089	+	+	X	
CAIX	Carbonic anhydrase 9 (CAIX)	Q16790	+	+	X	X
CASP-8	Caspase-8 (CASP-8)	Q14790	+	+	X	X
CCL17	C–C motif chemokine 17 (CCL17)	Q92583	+	+	X	X
CCL19	C–C motif chemokine 19 (CCL19)	Q99731	+	+	X	X
CCL20	C–C motif chemokine 20 (CCL20)	P78556	+	+	X	X
CCL23	C–C motif chemokine 23 (CCL23)	P55773	+	+	X	X
CCL3	C–C motif chemokine 3 (CCL3)	P10147	+	+	X	X
CCL4	C–C motif chemokine 4 (CCL4)	P13236	+	+	X	X
CD244	Natural killer cell receptor 2B4 (CD244)	Q9BZW8	+	+	X	X
CD27	CD27 antigen (CD27)	P26842	+	+	X	X
CD28	T-cell-specific surface glycoprotein CD28 (CD28)	P10747	+	+	X	X
CD4	T-cell surface glycoprotein CD4 (CD4)	P01730	+	+	X	X
CD40	CD40L receptor (CD40)	P25942	+	+	X	X
CD40-L	CD40 ligand (CD40-L)	P29965	+	+	X	X
CD5	T-cell surface glycoprotein CD5 (CD5)	P06127	+	+	X	X
CD70	CD70 antigen (CD70)	P32970	+	+	X	X
CD83	CD83 antigen (CD83)	Q01151	+	+	X	X
CD8A	T-cell surface glycoprotein CD8 alpha chain (CD8A)	P01732	+	+	X	X
CRTAM	Cytotoxic and regulatory T-cell molecule (CRTAM)	O95727	+	+	X	X
CSF-1	Macrophage colony-stimulating factor 1 (CSF-1)	P09603	+	+	X	X
CX3CL1	Fractalkine (CX3CL1)	P78423	+	+	X	X
CXCL1	C-X-C motif chemokine 1 (CXCL1)	P09341	+	+	X	X
CXCL10	C-X-C motif chemokine 10 (CXCL10)	P02778	+	+	X	X
CXCL11	C-X-C motif chemokine 11 (CXCL11)	O14625	+	+	X	X
CXCL12	Stromal cell-derived factor 1 (CXCL12)	P48061	+	+	X	X
CXCL13	C-X-C motif chemokine 13 (CXCL13)	O43927	+	+	X	X
CXCL5	C-X-C motif chemokine 5 (CXCL5)	P42830	+	+	X	X
CXCL9	C-X-C motif chemokine 9 (CXCL9)	Q07325	+	+	X	X
DCN	Decorin (DCN)	P07585	+	+	X	X
EGF	Pro-epidermal growth factor (EGF)	P01133	+	+	X	X
FASLG	Tumor necrosis factor ligand superfamily member 6 (FASLG)	P48023	+	+	X	X
FGF2^c^	Fibroblast growth factor 2 (FGF2)	P09038	+	+		X
Gal-1	Galectin-1 (Gal-1)	P09382	+	+	X	X
Gal-9	Galectin-9 (Gal-9)	O00182	+	+	X	X
GZMA	Granzyme A (GZMA)	P12544	+	+	X	X
GZMB	Granzyme B (GZMB)	P10144	+	+	X	X
GZMH	Granzyme H (GZMH)	P20718	+	+	X	X
HGF	Hepatocyte growth factor (HGF)	P14210	+	+	X	X
HO-1	Heme oxygenase 1 (HO-1)	P09601	+	+	X	X
ICOSLG	ICOS ligand (ICOSLG)	O75144	+	+	X	X
IL-1 alpha^c, d^	Interleukin-1 alpha (IL-1 alpha)	P01583	+	+		
IL-10	Interleukin-10 (IL10)	P22301	+	+	X	X
IL-12	Interleukin-12 (IL12)	P29459, P29460	+	+	X	X
IL-12RB1	Interleukin-12 receptor subunit beta-1 (IL12RB1)	P42701	+	+	X	X
IL-13^C,D^	Interleukin-13 (IL13)	P35225	+	+		
IL-18	Interleukin-18 (IL18)	Q14116	+	+	X	X
IL-2^c, e^	Interleukin-2 (IL2)	P60568	+	+		(X)
IL-33^c, d^	Interleukin-33 (IL33)	O95760	+	+		
IL-4	Interleukin-4 (IL4)	P05112	+	+	X	X
IL-5^d^	Interleukin-5 (IL5)	P05113	+	+		X
IL-6	Interleukin-6 (IL6)	P05231	+	+	X	X
IL-7	Interleukin-7 (IL7)	P13232	+	+	X	X
IL-8	Interleukin-8 (IL8)	P10145	+	+	X	X
KLRD1	Natural killer cells antigen CD94 (KLRD1)	Q13241	+	+	X	X
LAMP3	Lysosome-associated membrane glycoprotein 3 (LAMP3)	Q9UQV4	+	+	X	X
LAP TGF-beta-1	Latency-associated peptide transforming growth factor beta-1 (LAP TGF-beta-1)	P01137	+	+	X	X
MCP-1	C–C motif chemokine 2 (MCP-1)	P13500	+	+	X	X
MCP-2	C–C motif chemokine 8 (MCP-2)	P80075	+	+	X	X
MCP-3	C–C motif chemokine 7 (MCP-3)	P80098	+	+	X	X
MCP-4	C–C motif chemokine 13 (MCP-4)	Q99616	+	+	X	X
MIC-A/B	MHC class I polypeptide-related sequence A/B (MIC-A/B)	Q29983, Q29980	+	+	X	X
MMP-12	Macrophage metalloproteinase-12 (MMP12)	P39900	+	+	X	X
MMP-7	Matrix metalloproteinase-7 (MMP7)	P09237	+	+	X	X
NCR1	Natural cytotoxicity triggering receptor (NCR1)	O76036	+	+	X	X
NOS3	Nitric oxide synthase, endothelial (NOS3)	P29474	+	+	X	X
PDCD1	Programmed cell death protein 1 (PDCD1)	Q15116	+	+	X	X
PDGF subunit B	Platelet-derived growth factor subunit B (PDGF subunit B)	P01127	+	+	X	X
PD-L1	Programmed cell death 1 ligand 1 (PD-L1)	Q9NZQ7	+	+	X	X
PD-L2	Programmed cell death 1 ligand 2 (PD-L2)	Q9BQ51	+	+	X	X
PGF	Placenta growth factor (PGF)	P49763	+	+	X	X
PTN	Pleiotrophin (PTN)	P21246	+	+	X	X
TIE2	Angiopoietin-1 receptor (TIE2)	Q02763	+	+	X	X
TNFRSF12A	Tumor necrosis factor receptor superfamily member 12A (TNFRSF12A)	Q9NP84	+	+	X	X
TNFRSF21	Tumor necrosis factor receptor superfamily member 21 (TNFRSF21)	O75509	+	+	X	X
TNFRSF4	Tumor necrosis factor receptor superfamily member 4 (TNFRSF4)	P43489	+	+	X	X
TNFRSF9	Tumor necrosis factor receptor superfamily member 9 (TNFRSF9)	Q07011	+	+	X	X
TNFSF14	Tumor necrosis factor ligand superfamily member 14 (TNFSF14)	O43557	+	+	X	X
TRAIL	TNF-related apoptosis-inducing ligand (TRAIL)	P50591	+	+	X	X
TWEAK	Tumor necrosis factor ligand superfamily member 12 (TWEAK)	O43508	+	+	X	X
VEGFA	Vascular endothelial growth factor A (VEGFA)	P15692	+	+	X	X
VEGFR-2	Vascular endothelial growth factor receptor 2 (VEGFR-2)	P35968	+	+	X	X
IL-15^f,g^	Interleukin-15 (IL15)	P40933		+	X	(X)
KIR3DL1^f,g^	Killer cell immunoglobulin-like receptor 3DL1 (KIR3DL1)	P43629		+	X	(X)
LAG3^f,g^	Lymphocyte activation gene 3 protein (LAG3)	P18627		+	(X)	(X)
MUC-16^f,g^	Mucin-16 (MUC-16)	Q8WXI7		+	(X)	(X)
IFN- gamma^f,h^	Interferon gamma (IFN-gamma)	P01579	+	+	(X)	(X)
TNF^f,h^	Tumor necrosis factor (TNF)	P01375	+	+	(X)	(X)
IFN-beta^i^	Interferon beta	P01574	+			
IL-21^i^	Interleukin-21	Q9HBE4	+			
IL-35^i^	Interleukin-35	Q14213, P29459	+			
VEGFC^i^	Vascular endothelial growth factor C	P49767	+			

+ Protein included in the version of the Olink immune-oncology panel; *X* Protein included in all analyses; *(X)* Protein included in subset of analyses

^a^Serum-to-plasma variability investigated

^b^Temporal reproducibility and effect of storage investigated

^c^The protein failed quality control in at least one of five original studies using the newer version of the panel, and therefore not possible to assess interstudy variation

^d^More than 75% of the measurements below the LOD in serum or in both serum and EDTA plasma in serum-plasma sub-study and not included in analyses

^e^The protein failed quality control in the study using the older version of the panel, and it was only possible to assess interstudy variation for the protein between older and newer versions of the panel

^f^The ratio was not reported in the data provided by Olink, and the protein is therefore only evaluated on three criteria for plasma-to-serum ratio stability

^g^The protein was only included in the newer version and not possible to compare bridging sample stability between versions

^h^The protein assay changed significantly between older and newer versions, and therefore not possible to compare data between studies using different versions

^i^The protein were only included in the oldest version of the panel, which was only used in one study, and temporal reproducibility was not possible to assess

### Statistical analysis

For the analysis of serum-plasma variation, proteins with 75% or more of the measurement below the limit of detection (LOD) in either serum or plasma were excluded from further analysis. Table 3 shows the LOD for each protein assay as reported by Olink [13]. For all 12 patients and all remaining proteins, we calculated the ratio on a linear NPX scale as 2^(NPXserum − NPXplasma)^. Afterward, we estimated the geometric mean geometric standard deviation (SD), and geometric coefficient of variation (CV) of the serum-to-plasma ratio as well as median, 25% quartile (Q1), 75% quartile (Q3), and interquartile range (IQR).

**Table 3:** Protein serum and plasma levels and ratio stability.

Protein	sNPX^a^	pNPX^b^	LOD NPX^c^	pNPX below LOD (%)^d^	sNPX below LOD (%)^e^	Mean ratio^f^	CV% ratio^g^	Olink within range^h^	Correla-tion^i^	Concentration effect ^j^
Adenosine deaminase (ADA)	5.73	6.02	0.68	0	0	0,71	**25,39**	Yes	0.88	No
Adhesion G-protein coupled receptor G1 (ADGRG1)	2.95	2.67	0.68	0	0	1,21	10,20	Yes	0.97	No
Angiopoietin-1 (ANGPT1)	9.67	8.17	− 0.01	0	0	2,50	**29,41**	Yes	0.72	**Yes**
Angiopoietin-2 (ANGPT2)	6.87	6.78	0.33	0	0	1,13	19,77	Yes	0.88	No
Arginase-1 (ARG1)	4.60	4.23	4.32	50	33.33	1,42	**49,43**	Yes	**0.68**	No
Carbonic anhydrase 9 (CAIX)	6.14	5.87	1.04	0	0	1,20	5,69	Yes	0.99	No
Caspase-8 (CASP-8)	4.45	5.35	1.56	0	0	0,57	**37,88**	**No**	**0.34**	No
C–C motif chemokine 17 (CCL17)	10.93	10.19	1.35	0	0	1,47	**40,64**	**No**	**0.70**	No
C–C motif chemokine 19 (CCL19)	11.73	11.77	1.40	0	0	1,06	12,44	Yes	0.92	No
C–C motif chemokine 20 (CCL20)	8.58	9.18	1.12	0	0	0,62	16,25	Yes	0.98	No
C–C motif chemokine 23 (CCL23)	11.39	11.21	0.61	0	0	1,12	10,98	Yes	0.99	**Yes**
C–C motif chemokine 3 (CCL3)	7.65	7.05	0.48	0	0	1,42	14,97	Yes	0.73	No
C–C motif chemokine 4 (CCL4)	7.61	6.95	0.52	0	0	1,64	**20,66**	Yes	0.81	No
Natural killer cell receptor 2B4 (CD244)	6.93	7.17	1.67	0	0	0,76	**22,67**	Yes	**0.70**	No
CD27 antigen (CD27)	8.74	8.67	0.63	0	0	1,07	6,35	Yes	0.93	No
T-cell-specific surface glycoprotein CD28 (CD28)	0.87	0.76	0.29	8.33	0	1,07	12,70	Yes	0.81	No
T-cell surface glycoprotein CD4 (CD4)	4.37	4.22	0.62	0	0	1,06	9,17	**No**	0.95	No
CD40L receptor (CD40)	10.75	11.17	0.28	0	0	0,75	17,22	**No**	0.86	No
CD40 ligand (CD40-L)	7.73	6.75	2.18	0	0	1,42	**110,74**	**No**	**0.50**	No
T-cell surface glycoprotein CD5 (CD5)	6.41	6.39	0.56	0	0	1,03	6,61	Yes	0.91	No
CD70 antigen (CD70)	4.54	3.77	0.51	0	0	1,71	10,19	Yes	0.90	No
CD83 antigen (CD83)	3.24	3.11	0.44	0	0	1,06	9,06	Yes	0.80	No
T-cell surface glycoprotein CD8 alpha chain (CD8A)	10.03	10.04	0.21	0	0	1,01	8,44	Yes	0.97	No
Cytotoxic and regulatory T-cell molecule (CRTAM)	6.13	5.82	1.23	0	0	1,25	8,81	Yes	0.97	No
Macrophage colony-stimulating factor 1 (CSF-1)	10.53	10.50	1.35	0	0	1,04	5,71	Yes	0.91	No
Fractalkine (CX3CL1)	4.74	4.32	0.43	0	0	1,28	8,71	Yes	0.99	No
C-X-C motif chemokine 1 (CXCL1)	10.35	10.31	0.99	0	0	1,04	**61,37**	Yes	**0.70**	**Yes**
C-X-C motif chemokine 10 (CXCL10)	10.19	10.56	1.01	0	0	0,85	19,48	Yes	0.94	No
C-X-C motif chemokine 11 (CXCL11)	9.59	10.37	0.77	0	0	0,61	**51,33**	**No**	0.76	No
Stromal cell-derived factor 1 (CXCL12)	1.92	2.28	0.92	0	0	0,81	17,62	Yes	0.84	No
C-X-C motif chemokine 13 (CXCL13)	9.81	9.60	1.43	0	0	1,08	**23,29**	Yes	0.94	**Yes**
C-X-C motif chemokine 5 (CXCL5)	12.59	12.37	1.39	0	0	1,07	**48,26**	**No**	**0.59**	No
C-X-C motif chemokine 9 (CXCL9)	8.31	8.47	0.76	0	0	0,96	10,60	Yes	0.80	No
Decorin (DCN)	4.89	4.52	0.92	0	0	1,30	8,46	Yes	**0.66**	No
Pro-epidermal growth factor (EGF)	9.28	8.45	0.69	0	0	1,29	**123,45**	**No**	**0.36**	No
Tumor necrosis factor ligand superfamily member 6 (FASLG)	7.07	7.05	0.69	0	0	1,05	8,75	Yes	0.92	No
Fibroblast growth factor 2 (FGF2)^#^	0.43	2.74	0.53	8.33	75	NA	NA	NA	NA	NA
Galectin-1 (Gal-1)	7.08	7.11	1.19	0	0	0,96	8,27	Yes	0.88	No
Galectin-9 (Gal-9)	8.80	8.78	0.24	0	0	1,03	5,89	Yes	0.92	No
Granzyme A (GZMA)	7.47	7.35	0.53	0	0	1,05	19,82	Yes	0.74	No
Granzyme B (GZMB)	3.40	4.01	0.28	0	0	0,79	**53,05**	Yes	**0.57**	No
Granzyme H (GZMH)	4.56	4.76	1.46	0	0	0,89	19,02	Yes	0.88	No
Hepatocyte growth factor (HGF)	10.22	9.45	0.65	0	0	1,71	**26,41**	Yes	0.86	No
Heme oxygenase 1 (HO-1)	12.66	12.50	4.41	0	0	1,04	8,04	Yes	0.97	No
ICOS ligand (ICOSLG)	6.25	5.74	0.39	0	0	1,40	7,82	Yes	0.83	No
Interferon gamma (IFN-gamma)^*^	7.82	7.64	1.92	0	0	1,07	9,81	NA	0.99	No
Interleukin-1 alpha (IL-1 alpha)^#^	0.22	0.48	2.24	100	100	0,95	36,09	NA	NA	NA
Interleukin-10 (IL10)	4.49	4.39	1.67	0	0	1,15	13,41	Yes	0.95	No
Interleukin-12 (IL12)	7.59	7.44	1.50	0	0	1,12	10,68	Yes	0.95	No
Interleukin-12 receptor subunit beta-1 (IL12RB1)	2.52	2.49	1.26	0	0	1,05	12,94	Yes	0.90	No
Interleukin-13 (IL13)^#^	1.63	1.60	2.49	91.67	91.67	NA	NA	NA	NA	NA
Interleukin-15 (IL15)^*^	5.43	5.29	2.29	0	0	1,12	9,18	NA	0.91	No
Interleukin-18 (IL18)	10.05	10.20	1.16	0	0	0,95	18,08	Yes	0.93	No
Interleukin-2 (IL2)^#^	1.35	1.30	2.27	100	100	NA	NA	NA	NA	NA
Interleukin-33 (IL33)^#^	− 0.57	− 0.42	− 0.06	91.67	100	NA	NA	NA	NA	NA
Interleukin-4 (IL4)	1.05	1.36	1.08	16.67	50	0,86	**28,44**	Yes	**0.36**	No
Interleukin-5 (IL5)^#^	1.03	0.99	1.44	66.67	75	NA	NA	NA	NA	NA
Interleukin-6 (IL6)	4.82	4.87	1.05	0	0	1,08	10,86	Yes	1.00	No
Interleukin-7 (IL7)	6.80	6.13	0.71	0	0	1,62	**47,62**	Yes	**0.53**	No
Interleukin-8 (IL8)	8.07	6.99	2.24	0	0	2,70	**37,77**	Yes	0.92	No
Killer cell immunoglobulin-like receptor 3DL1 (KIR3DL1)^*^	2.46	2.50	1.29	0	8.33	0,98	16,28	NA	0.96	No
Natural killer cells antigen CD94 (KLRD1)	7.19	7.03	0.25	0	0	1,16	6,72	Yes	0.99	No
Lymphocyte activation gene 3 protein (LAG3)^*^	5.81	5.50	1.05	0	0	1,22	7,69	NA	0.97	No
Lysosome-associated membrane glycoprotein 3 (LAMP3)	5.38	5.26	0.66	0	0	1,14	7,93	Yes	0.97	No
Latency-associated peptide transforming growth factor beta-1 (LAP TGF-beta-1)	10.26	10.10	1.67	0	0	1,01	**26,78**	**No**	0.87	No
C–C motif chemokine 2 (MCP-1)	12.01	11.24	0.72	0	0	1,92	**20,32**	**No**	0.80	No
C–C motif chemokine 8 (MCP-2)	8.96	8.38	0.79	0	0	1,51	**23,85**	Yes	0.76	No
C–C motif chemokine 7 (MCP-3)	3.12	2.26	0.91	0	0	1,86	**21,03**	Yes	0.71	No
C–C motif chemokine 13 (MCP-4)	11.19	10.57	0.39	0	0	1,53	**35,37**	Yes	**0.53**	No
MHC class I polypeptide-related sequence A/B (MIC-A/B)	6.16	6.03	0.78	0	0	1,07	7,49	Yes	0.93	No
Macrophage metalloproteinase-12 (MMP12)	8.97	8.43	0.42	0	0	1,31	**20,91**	Yes	0.97	**Yes**
Matrix metalloproteinase-7 (MMP7)	13.04	11.49	4.09	0	0	3,01	19,69	**No**	**0.55**	No
Mucin-16 (MUC-16)^*^	5.59	5.52	0.20	0	0	1,04	8,40	NA	0.99	No
Natural cytotoxicity triggering receptor (NCR1)	4.09	4.16	1.34	0	0	1,10	13,09	Yes	0.89	No
Nitric oxide synthase, endothelial (NOS3)	2.55	3.34	1.97	0	16.67	0,63	**30,82**	Yes	0.72	No
Programmed cell death protein 1 (PDCD1)	5.67	5.65	2.53	0	0	1,00	8,44	Yes	0.96	No
Platelet-derived growth factor subunit B (PDGF subunit B)	10.65	10.38	0.73	0	0	1,26	**28,30**	Yes	-**0.52**	**Yes**
Programmed cell death 1 ligand 1 (PD-L1)	6.74	7.03	1.85	0	0	0,84	13,84	Yes	0.93	No
Programmed cell death 1 ligand 2 (PD-L2)	3.72	3.61	0.93	0	0	1,13	6,24	Yes	0.96	No
Placenta growth factor (PGF)	9.17	9.09	0.75	0	0	1,09	7,17	Yes	0.94	No
Pleiotrophin (PTN)	1.47	2.93	1.14	0	33.33	0,41	**41,52**	Yes	**0.44**	No
Angiopoietin-1 receptor (TIE2)	7.77	7.65	0.54	0	0	1,05	6,87	Yes	0.97	No
Tumor necrosis factor (TNF)^*^	5.23	5.20	2.28	0	0	1,00	12,36	NA	0.87	No
Tumor necrosis factor receptor superfamily member 12A (TNFRSF12A)	6.33	6.13	-0.10	0	0	1,27	6,94	**No**	0.99	No
Tumor necrosis factor receptor superfamily member 21 (TNFRSF21)	7.86	7.77	-0.39	0	0	1,10	6,39	Yes	0.95	No
Tumor necrosis factor receptor superfamily member 4 (TNFRSF4)	6.89	6.78	0.82	0	0	1,13	8,21	Yes	0.96	No
Tumor necrosis factor receptor superfamily member 9 (TNFRSF9)	7.44	7.19	0.81	0	0	1,13	5,58	Yes	0.99	No
Tumor necrosis factor ligand superfamily member 14 (TNFSF14)	7.07	6.20	0.70	0	0	2,07	**39,87**	Yes	0.80	No
TNF-related apoptosis-inducing ligand (TRAIL)	8.48	8.12	0.53	0	0	1,32	10,56	Yes	0.73	No
Tumor necrosis factor ligand superfamily member 12 (TWEAK)	8.58	8.20	-0.06	0	0	1,46	**23,29**	Yes	**0.64**	No
Vascular endothelial growth factor A (VEGFA)	10.80	10.33	0.37	0	0	1,45	**24,07**	Yes	0.93	No
Vascular endothelial growth factor receptor 2 (VEGFR-2)	8.20	8.12	0.54	0	0	1,07	6,52	Yes	0.92	No

Values highlighted in bold indicate criteria not fulfilled

^a^Median serum normalized protein expression (NPX) value

^b^Median plasma NPX value

^c^Limit of detection (LOD) reported as NPX value

^d^Plasma samples below LOD

^e^Serum samples below LOD

^f^Geometric mean of serum to plasma ratio

^g^Geometric coefficient of variation (CV) in percentage

^h^Ratio reported by Olink [13] within range observed in our study

^i^Correlation coefficient between serum and plasma NPX

^j^Association between concentration and protein ratio on Passing-Bablok regression or Bland–Altman plots

^g^^−^^j^Values not fulfilling the four criteria indicated in bold

^#^Not evaluated because of to many samples below LOD

^*^Olink did not report serum-plasma ratios

To estimate variation between serum and EDTA plasma, we calculated the Spearman correlation coefficient plotting serum NPX vs. EDTA plasma NPX. To evaluate a potential association between concentration and ratio, a Passing-Bablok regression of EDTA plasma NPX vs. serum NPX was performed. For each protein, slope and intercept with 95% confidence interval (CI) were calculated and regression line plotted against an identity line. Likewise, modified Bland–Altman plots were generated for all proteins, plotting serum-plasma ratio against the mean plasma-serum concentration, and fitted linear regression using the lm function (*stat* package). Lastly, we tested the effect of bilirubin level on the ratio using linear regression. A 2-sided *p*-value of 0.05 was used as the level of significance for regression analyses.

For each protein, the reproducibility of the serum-plasma variation was evaluated using five criteria:

1. CV of the serum-plasma ratio was less than 20%. The threshold correspond to the highest inter and intraassay CV for the protein assays used in the panel [13]
2. The ratio reported in the Olink validation data document [13] was within range (Q1 − 1.5 × IQR, and Q3 + 1.5 IQR) observed in our study
3. The correlation coefficient was above 0.7
4. No association between concentration and ratio was identified. A protein ratio was defined as having an association if the slope of the Passing-Bablok regression was significantly different from 1 or a significant association was observed on Bland–Altman plots

To assess the temporal reproducibility and effect of storage, the inter-study, and the intra-study, inter-assay variation was quantified by calculating mean NPX, SD, and CV for each protein and bridging sample on a linear NPX (2^NPX^) scale. Afterwards, an interstudy normalization was performed following manufactures recommendation using the bridging samples with plate from BX0150 as reference. The median linear NPX per study per target for all bridging samples from all plates (*n* = 36) combined was also calculated. Inter-study variation was also assessed using Pearson correlation between median NPX in all studies. The median was chosen because median NPX is recommended to be used for bridging. Inter-study variation was also compared using Bland–Altman plots, plotting the difference in median concentration between studies against the mean of the two median concentrations.

Statistical analyses were performed using R (R Core Team (2019. R: A language and environment for statistical computing. R Foundation for Statistical Computing, Vienna, Austria).

## Results

The study investigated the serum-to-plasma ratio for 86 proteins using matched serum and plasma samples from 12 patients. Six proteins had more than 75% of the measurements below the LOD in serum or in both serum and EDTA plasma (IL1 alpha, IL2, IL5, IL13, IL33 and fibroblast growth factor 2 (FGF2)). The temporal reproducibility and the effect of storage were assessed using data from 6 studies. In total, we had data from 36 plates where the same serum bridging samples had been analyzed. On 31 plates, all 8 serum samples had been included and on the last 5 between 6 and 7 serum samples were used (Table 1). The temporal reproducibility was assessed for 81 proteins across all 6 studies after removing proteins where the assay was changed between versions, not included in both versions, or failed quality control in at least one study. For 88 proteins, it was possible to compare results across the five studies using the newer version of the Olink I-O. Table 2 lists all proteins included in each sub study.

### Differences between protein levels in serum and plasma samples

**Fig. 1:**
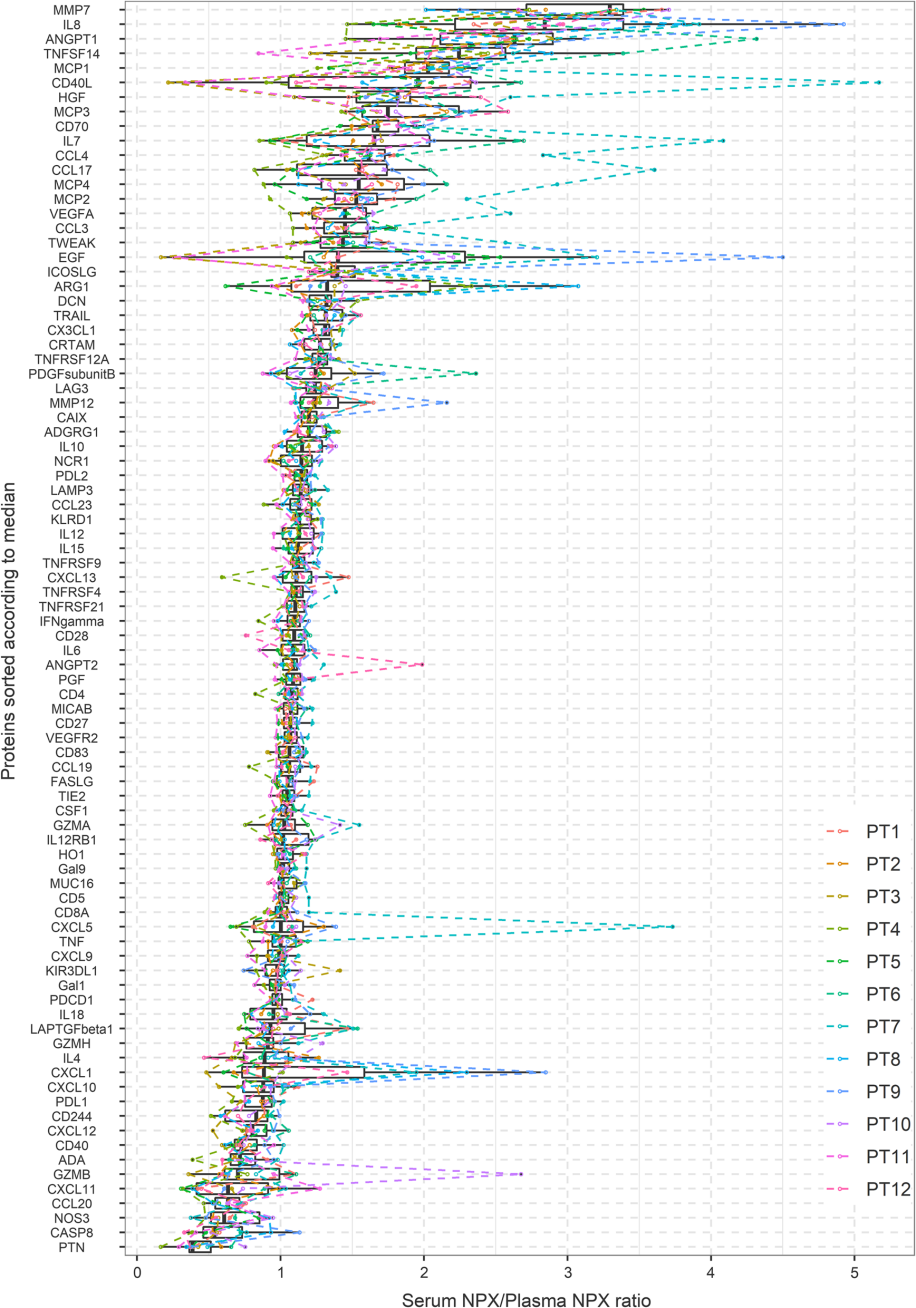
Boxplot of serum-to-plasma ratio on linear normalized protein expression (NPX) scale for the 86 proteins with more than 75% of samples over limit of detection. Proteins sorted according to median serum-to-plasma ratio. Samples from each patient connected with dashed lines

**Fig. 2:**
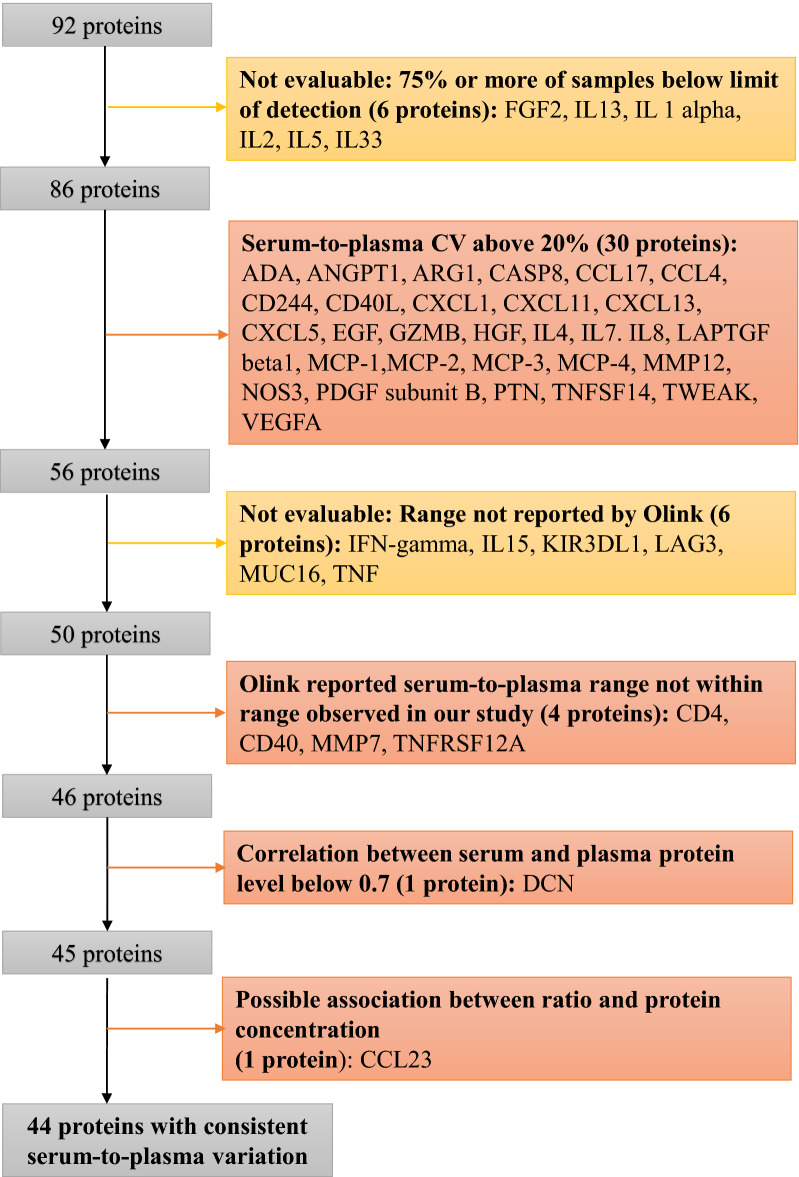
Consort diagram

For most proteins, the serum and EDTA plasma concentrations were similar (Additional file 1: Figure S1). Of the 86 proteins included in the sub-study, the mean serum-to-EDTA plasma ratio ranged from 0.41 to 3.01 (Table 3). Fifty-two proteins had a minor variation, with a mean ratio from 0.80 to 1.20, and the variation seemed to be randomly distributed between samples (Fig. 1). For the 12 samples, bilirubin ranged from 5 to 812 with two patients having a higher bilirubin than 200. There was no significant association between serum-to-EDTA plasma ratio and bilirubin after adjusting for multiple comparisons. Next, we evaluated the reproducibility of the ratio using the above-mentioned criteria. Figure 2 illustrates the consort diagram showing which proteins fulfilled each criterion.

Criterion 1 (CV of the serum – EDTA plasma ratio < 20%): The geometric CV of the ratio ranged from 5.58% to 123.45%. Thirty proteins had a CV of more than 20%. Of note, most of these proteins had either a high or low median NPX in both serum and EDTA plasma (Additional file 1: Figure S2), and the highest CVs were observed for proteins with high or low ratios.

**Fig. 3:**
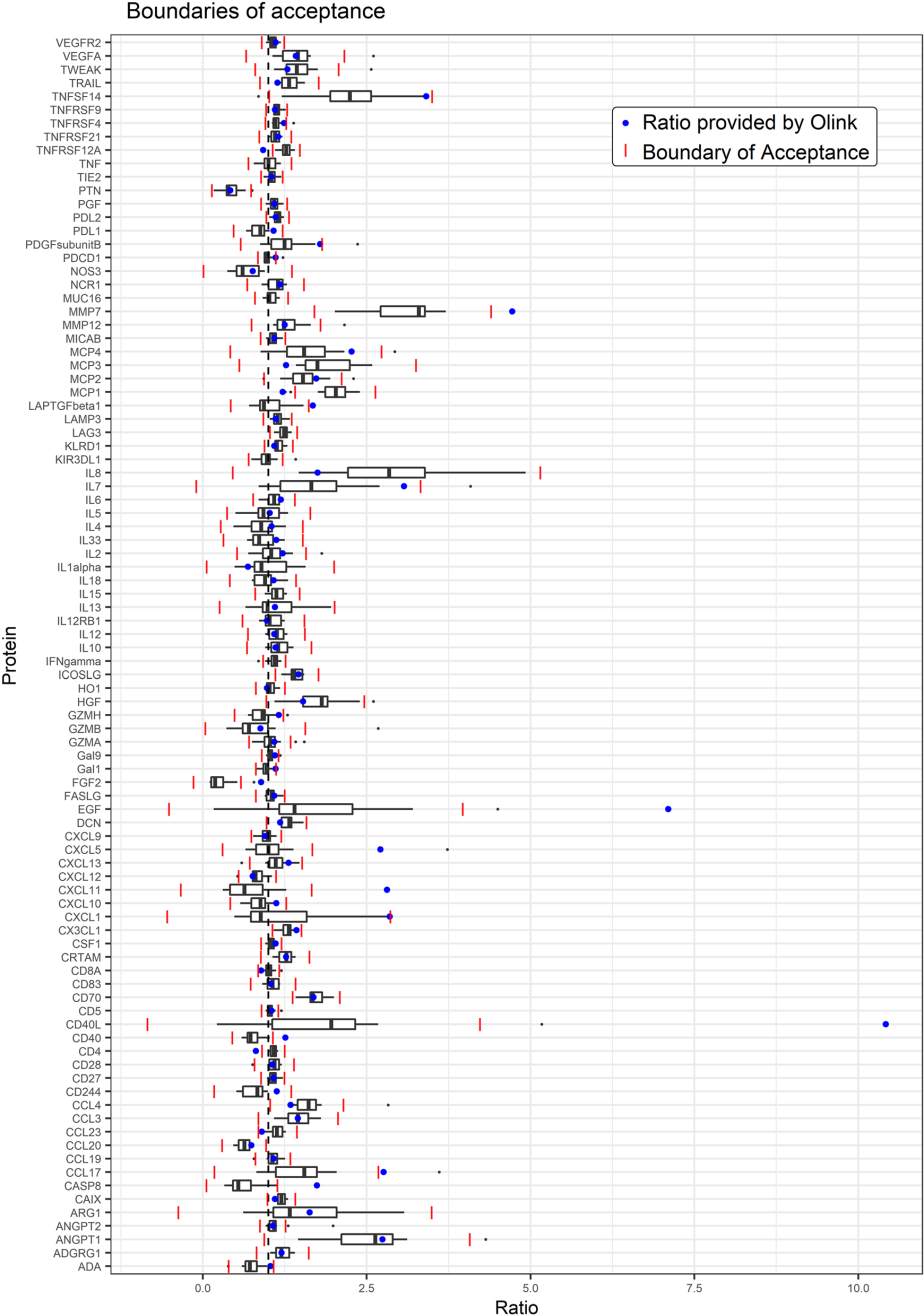
Serum-to-plasma ratio in our study and as reported by Olink on linear normalized protein expression (NPX) scale. Red lines indicate boundaries of acceptance (25% quartile (Q) + 1.5 interquartile range (IQR) and 75% Q + 1.5IQR). Blue points indicate serum-to-plasma ratio reported by Olink. Data were not reported by Olink for 6 proteins: KIR3DL1, MUC-16, TNF, IL15, LAG3, IFN-gamma

Criterion 2 (The ratio between serum and EDTA plasma reported by Olink Proteomics [13] is within range (Q1—1.5 × IQR and Q3 + 1.5 × IQR) of the observed in our study: Of the 56 proteins with a reasonable CV, there were four proteins where Olink reported a ratio that was markedly different from the range observed in our study [T-cell surface glycoprotein CD4 (CD4), CD40 ligand receptor (CD40), C–C motif chemokine 2 (MCP-1), matrix metalloproteinase-7 (MMP7), tumor necrosis factor receptor superfamily member 12A (TNFRSF12A)]. Seven proteins had both a high CV in our study and a ratio reported by Olink that was outside the range in study [pro-epidermal growth factor (EGF), CD40 ligand (CD40-L), C-X-C motif chemokine (CXCL) 5, CXCL11, C–C motif chemokine (CCL) 17, caspase-8 (CASP-8), latency-associated peptide transforming growth factor beta-1 (LAP TGF-beta-1)] (Fig. 3). The ratio was not reported for six proteins (KIR3DL1, MUC-16, TNF, IL15, LAG3, IFN-gamma) in the data provided by Olink, and it is therefore not possible to compare the results for these proteins. Of note, all six passed the other three criteria.

Criterion 3 (The correlation coefficient found in regression analysis was above 0.7): Seventeen proteins had a poor correlation between results obtained in serum and EDTA plasma, with a correlation coefficient (*r*) ≤ 0.7. However, 16 of these also had a poor CV or Olink data were not within range, and therefore only one protein—decorin (DCN), was removed due to this criterion. Correlation diagrams for all proteins are shown in Additional file 1: Figure S3.

Criterion 4 (No association between concentration and ratio): For six proteins we identified an association between ratio and concentration. Five proteins had a slope of the Passing-Bablok regression which was significantly lower than 1 (platelet-derived growth factor subunit B (PDGF subunit B), angiopoietin-1 (ANGPT1), CXCL13, CCL23, MMP12), and for one protein the slope was significantly higher (CXCL1) (Additional file 1: Figure S3). A similar result was seen using Bland–Altman plots, where 5 of the 6 proteins (CXCL1, PDGF subunit B, ANGPT1, CXCL13, CCL23) had a significant association between ratio and protein concentration using linear regression (Additional file 1: Fig. S4). Five of the six proteins were already removed due to one of the prior criteria. Only CCL23 was removed due to an association between concentration and ratio.

**Fig. 4:**
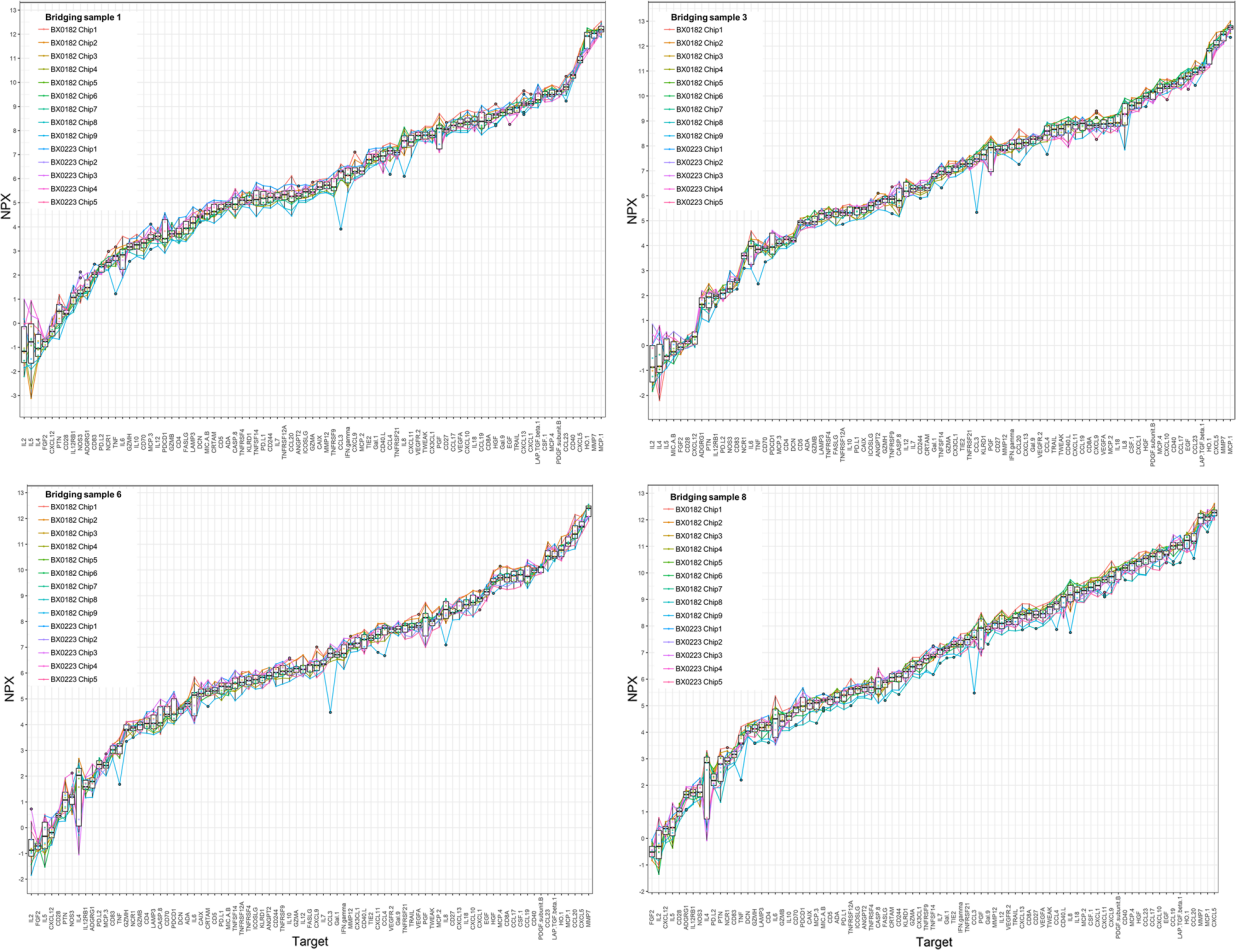
Normalized protein expression for four bridging samples in BX0223 and BX0182. Boxplots showing results of all plates (chips) for each protein and bridging sample as measured in BX0223 (number of assays = 5) and BX0182 (number of assays = 9). Each graph represents results from one bridging sample. Results from each assay connected with colored lines

Of the 92 proteins included in the Olink I-O panel, 80 proteins were possible to evaluate on all four criteria for serum-to-EDTA plasma variation reproducibility. Of these, 44 proteins fulfilled all four and 36 failed on one or more of the criteria (Table 3 and Fig. 2).

### Temporal reproducibility and effect of storage

In general, we observed a similar intra-study inter-assay variation based on the serum bridging samples in the three studies that used three or more plates and in those that used the newest Olink panel (BX0182, BX0188, and BX0223). For these studies, 88 proteins were in common after removing four (IL1 alpha, IL13, IL33, and ARG1) that did not pass quality control. Very few proteins had a variation higher than 1 NPX, and only a few random outliers were observed. Figure 4 shows NPX values for all assays in BX0223 and BX0182 for 4 of the bridging samples. The three studies had a similar intra-study inter-assay variation. The mean CV was 11.3% in BX0188, 16.4% in BX0182, and 12.3% in BX0223. For the most recent of the three studies (BX0223), where bridging samples had been stored the longest, only seven proteins had a CV higher than 20% (IL2, IL5, CD40-L, endothelial nitric oxide synthase (NOS3), natural killer cells antigen CD94 (KIR3DL), pleiotrophin (PTN), IL4), but except for CD40-L, all were characterized by low NPX values, with several being below LOD. For the oldest study (BX0144) that used an earlier version of the Olink I-O panel, the inter-assay variation was higher. When comparing all 91 proteins used in the panel and passed quality control in the study, the interstudy CV was 26.0%, and when only the 81 proteins in common between all studies were included, the CV was 22.6%.

In all studies, pooled serum samples had been included on all plates as recommended by the manufacturer. For all studies the inter-assay intra-study variation based on the serum bridging samples were similar or better than the inter-assay variation based on pooled serum samples (36% for BX0144, 17% for BX0182, 10% for BX0188, and 17% for BX0223).

**Fig. 5:**
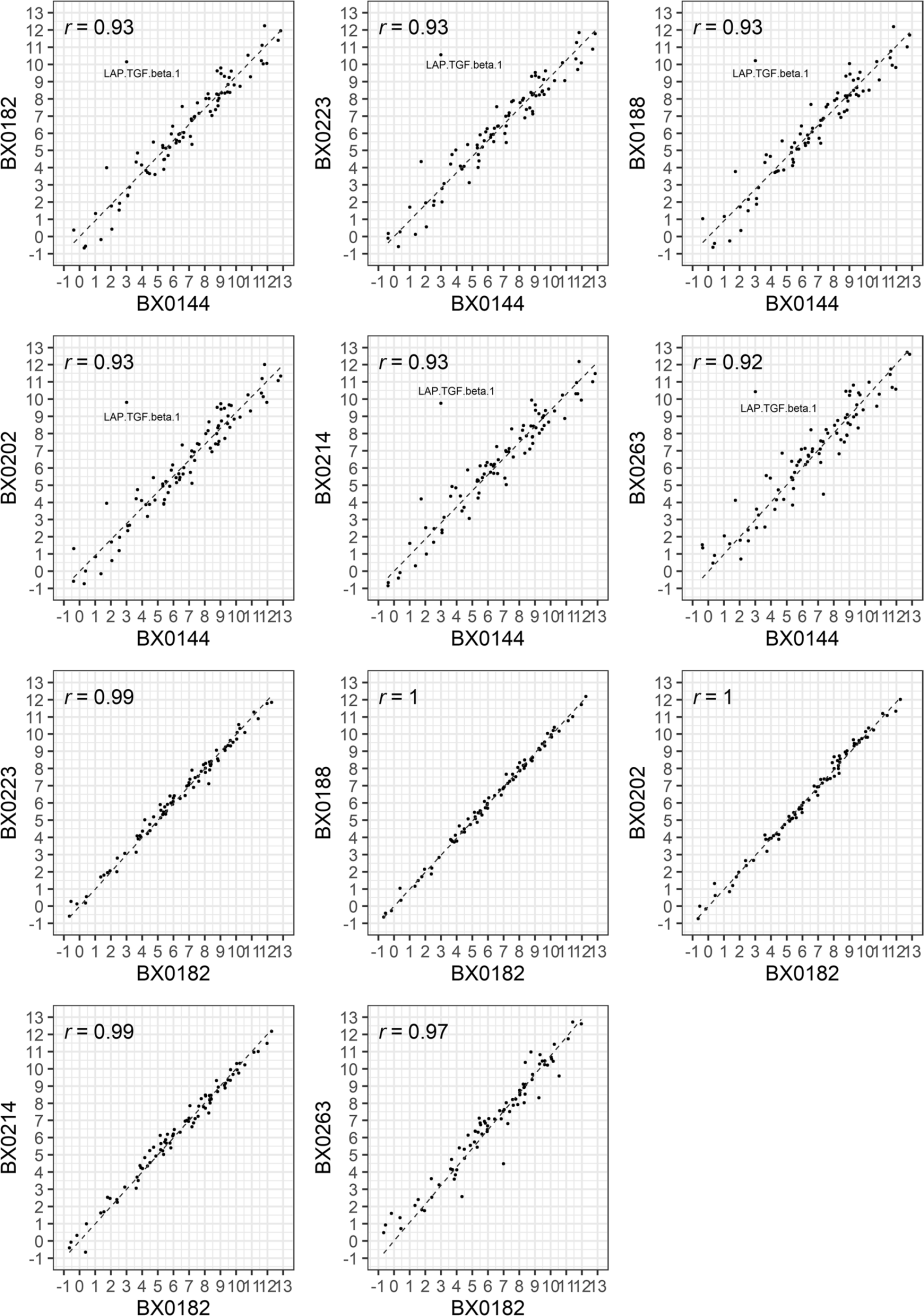
Correlation diagram comparing bridging sample results between studies using median NPX for all proteins in common between BX0144 and BX0182 and the 5 other studies. In each plot, a dot denotes a given protein. Latency-associated peptide transforming growth factor beta-1 (LAP TGF-beta-1) highlighted as a significant outlier

The mean inter-study CV across all studies based on 81 proteins measured using the serum bridging samples was 41.3% (range: 17.6–109.9%). After normalization between studies the mean inter-study CV decreased to 26.2% (range 11.3–108.5%). The inter-study variation was significantly lower after removing BX0144, which used the oldest version of the panel (pre-normalization mean CV = 26.9%, range 13.6–73.8%, post-normalization CV = 18.9%, range 7.3–66.1%). The pre-normalized CV was further reduced in the studies that only used v. 3111 (CV = 19.7%, range: 11.8–59.1%), but not the post-normalization CV (mean = 19.05%, range 7.5–67.2%). For the five studies using v. 3111, 3 proteins had a CV higher than 40% [IL4 (CV = 58.9%), MHC class I polypeptide-related sequence A/B (MIC A/B) (CV = 51.7%), and IL5 (CV = 41.6%)], and all three had low median NPX values and/or several samples below LOD. Pearson correlation analysis (Fig. 5) showed a high correlation (*r* ≥ 0.97) between results obtained in studies that used the newer version of the panel (v. 3111 and v. 3112) and highest for studies using the same version. The correlation decreased (*r* = 0.92–0.93) when results from BX0144 were compared with the six other studies, including the 81 proteins in common. One protein, LAP.TGF.beta.1, had a large difference between BX0144 (NPX = 2.99) and the other studies (NPX: 9.76–10.56). When LAP.TGF.beta.1 was removed, the correlation between the studies improved (*r* = 0.95–0.97). The Bland–Altman plots showed a similar pattern, with a smaller variation between studies using the same panel but a larger variation between the BX0144 and the other studies. Of note, no trends were observed in the plots (Additional file 1 Figure S5).

## Discussion

To avoid any effect on the results, it is generally recommended to use the same type of blood matrices (e.g. serum or EDTA plasma) in all phases of circulating protein biomarker studies [14, 15]. However, obtaining the same matrices from all patients in a study may not always be feasible or even possible. Limited data exist regarding serum-to-EDTA plasma variation for the PEA-based protein analyses, including the Olink® I-O panel [13]. In the present study, we evaluated 80 of 92 proteins based on four criteria. Six proteins were not evaluated due to a high number of samples below the LOD, and for 6 other proteins, we were only able to evaluate the serum-to-plasma ratio for three of four criteria. Of the 80 fully evaluated proteins, 36 (45%) had a high variation of the serum and EDTA plasma ratio, a poor correlation between serum and plasma values, or a possible effect of concentration on the ratio. Some of the variability might be explained by some proteins having a concentration at the lower or upper limit of quantification of the assay. A different explanation might be a difference in pre-centrifuge storage time, which might have led to degradation of proteins or cellular leak [16, 17].

Previously, Shen et al. investigated the effect of storage time and temperature on protein concentration in EDTA plasma using Olink® Cardiology and an inflammation panel including 50 of the proteins analyzed in our study. Compared with immediate centrifuging, they identified a significant change in protein concentration in 5% and 10% of the proteins stored at 22 °C for 1 and 3 h [17]. Since we stored the blood for up to two hours at room temperature before centrifuging the tubes, this might have impacted the protein results of our study. Interestingly, the proteins most significantly affected by pre-centrifuging conditions were CD40-L, EGF, PDGF-subunit B, CXCL5, and MMP7 [17]. All these proteins had a high variation or poor correlation between serum and plasma. It was not possible in our study to test whether we would obtain different ratios if pre-centrifuge conditions or storage times were changed because we used a SOP suitable for use in daily clinical practice.

Our study showed that bridging samples can be stored and used for over three years and with different versions of the same PEA panel and give compatible results. The inter-assay CV of the bridging samples was within the inter-assay variation based on sample controls, and for the studies using the newer Olink® I-O panel, our results are also compatible with the validation data document provided by Olink® [13]. The high inter-assay variation in the oldest study (BX0144), using the older version 3101 of the Olink® I-O panel), might be due to it using significantly more plates, which meant that BX0144 had to be run over several days. The inter-study variation was higher than the intra-study, especially when comparing data obtained using the old and new panel. Likewise, the correlation between results obtained using different panels was also poorer than the correlation between studies using the same panel. Some of these results might be explained by a higher inter-assay variation in the oldest study (BX0144), but they might also be due to version changes by Olink®. For the study (BX0263) using the newest panel (v. 3112), we only used one plate, and therefore it was not possible to assess intra-study variation, and comparisons between BX0263 and the other studies are too uncertain for firm conclusions.

Interestingly, most of the evaluated proteins with a high inter-assay CV were classified as having an inconsistent serum-to-EDTA plasma ratio, which points to these proteins being volatile and susceptible to changes due to pre-centrifuge preparation or preanalytical conditions. Likewise, our results might have been affected by the storage time of the samples for 3–51 months at − 80 °C. Enroth et al. investigated the effect of storage time on EDTA plasma concentration of proteins measured with PEA technology and identified 18 of 102 proteins where the protein level was affected by a storage time of up to 30 years, with 5–35% of the variation being explained by storage [18].

In summary, for 44 of 80 proteins included on the Olink® I-O panel and evaluated in the present study, the results obtained using serum and EDTA plasma were well correlated, and the serum-to-plasma ratio remained constant. Furthermore, we showed that bridging samples can be stored for several years and used on different versions of the same PEA panel with compatible results.

## Supplementary Information

**Additional file 1: Figure S1**: Boxplot of median serum and plasma concentration. **Figure S2**: Serum-to-plasma ratio according to median serum concentration. **Figure S3**: Correlation diagram and Passing-Bablok regression of serum vs. plasma value for all proteins for all 92 proteins. **Figure S4**: Bland-Altman plots of serum-to-plasma ratios. **Figure S5**: Bland–Altman plot comparing BX0144 with all other studies, and BX0182 with BX0188, BX0202, BX0214, BX0223, and BX0263.

## Data Availability

The datasets used and/or analyzed during the current study are available from the corresponding author on reasonable request.

## Abbreviations

PEA: Proximity extension assay
EDTA: Ethylenediamine tetraacetic acid
I-O: Immuno-oncology
PCR: Polymerase chain reaction
NPX: Normalized protein expression
SOP: Standard operating procedure
IL: Interleukin
IFN: Interferon
VEGFC: Vascular endothelial growth factor C
LAG3: Lymphocyte activation gene 3 protein
MUC-16: Mucin-16
KIR3DL1: Killer cell immunoglobulin-like receptor 3DL1
TNF: Tumor necrosis factor
ARG-1: Arginase-1
LOD: Limit of detection
SD: Standard deviation
Q: Quartile
IQR: Interquartile range
CV: Coefficient of variation
FGF2: Fibroblast growth factor 2
CD28: T-cell-specific surface glycoprotein CD28
CD40-L: CD40 ligand
PTN: Pleiotrophin
GZMB: Granzyme B
MCP-3: C–C motif chemokine 7
EGF: Pro-epidermal growth factor
CXCL12: Stromal cell-derived factor 1
NOS3: Nitric oxide synthase: endothelial
CASP-8: Caspase-8
CCL: C–C motif chemokine
CD4: T-cell surface glycoprotein
CD40: CD40 ligand receptor
CXCL: C-X-C motif chemokine
LAP TGF-beta-1: Latency-associated peptide transforming growth factor beta-1
MCP-1: C–C motif chemokine 2
MMP-7: Matrix metalloproteinase-7
TNFRSF12A: Tumor necrosis factor receptor superfamily member 12A
PDGF subunit B: Platelet-derived growth factor subunit B
ANGPT1: Angiopoietin-1
MIC A/B: MHC class I polypeptide-related sequence A/B
