# Supplementary material for: Determination of temporal reproducibility and variability of cancer biomarkers in serum and EDTA plasma samples using a proximity extension assay
Source: Clin Proteomics. 2022 Nov 15;19:39. doi: 10.1186/s12014-022-09380-y (PMC9664820; doi:10.1186/s12014-022-09380-y)
Supplement: Supplementary file 1 — Additional file 1: Figure S1: Boxplot of median serum and plasma concentration. Figure S2: Serum-to-plasma ratio according to median serum concentration. Figure S3: Correlation diagram and Passing-Bablok regression of serum vs. plasma value for all proteins for all 92 proteins. Figure S4: Bland-Altman plots of serum-to-plasma ratios. Figure S5: Bland–Altman plot comparing BX0144 with all other studies, and BX0182 with BX0188, BX0202, BX0214, BX0223, and BX0263. [file 12014_2022_9380_MOESM1_ESM.docx]

**Supplemental Tables**

**Supplemental figures**

**Supplemental Figure 1**: **Boxplot of median serum and plasma concentration**

Serum and plasma level of the 92 proteins for the 12 patients. Plot includes measurements below limit of detection. Proteins sorted according to median concentration in all samples. NPX = normalized protein expression.


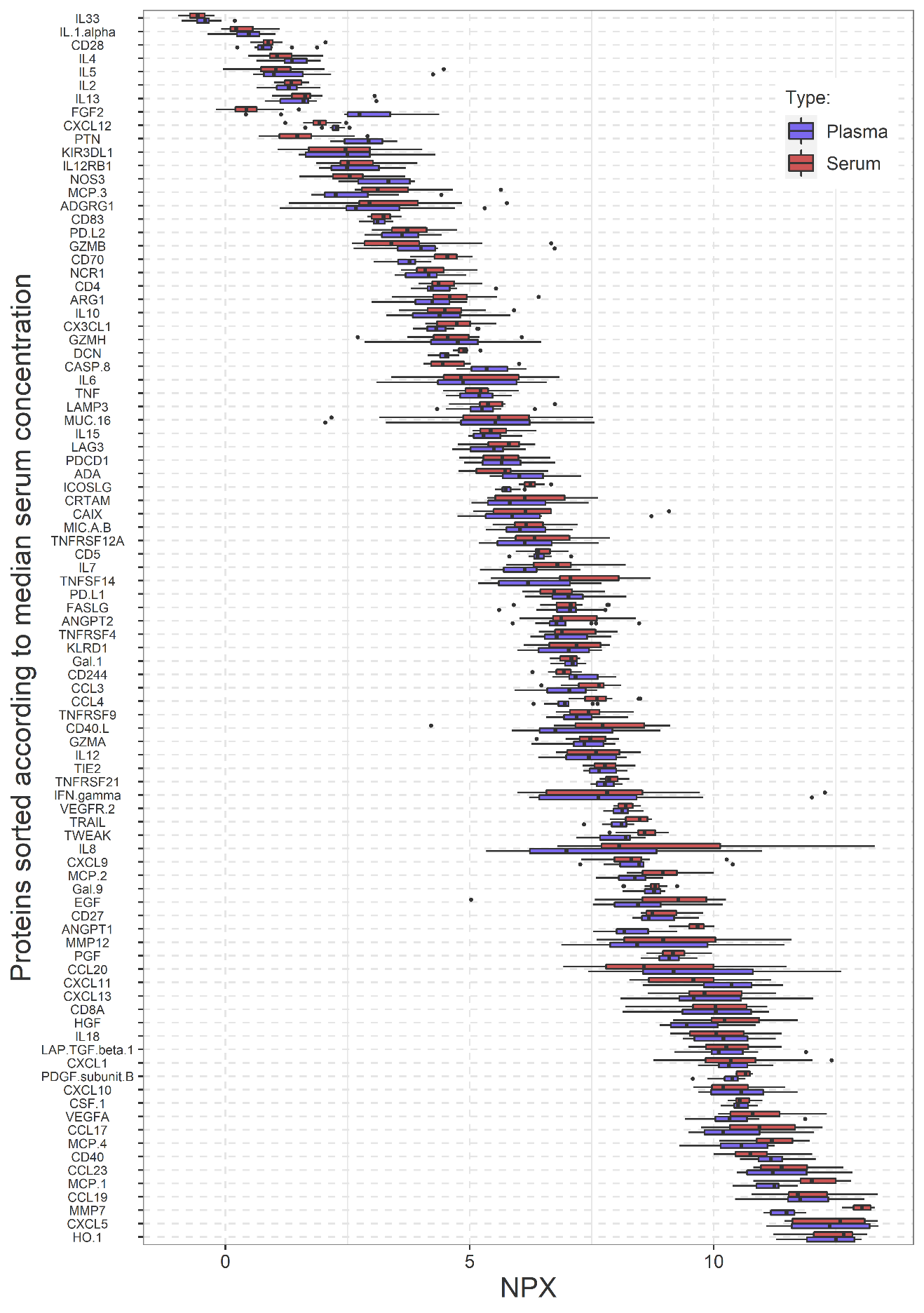


**Supplemental Figure 2: Serum-to-plasma ratio according to median serum concentration**

Boxplot of serum-to-plasma ratio for the 86 proteins with more than 75% of samples over limit of detection. Proteins sorted according to median serum concentration. PT = patient


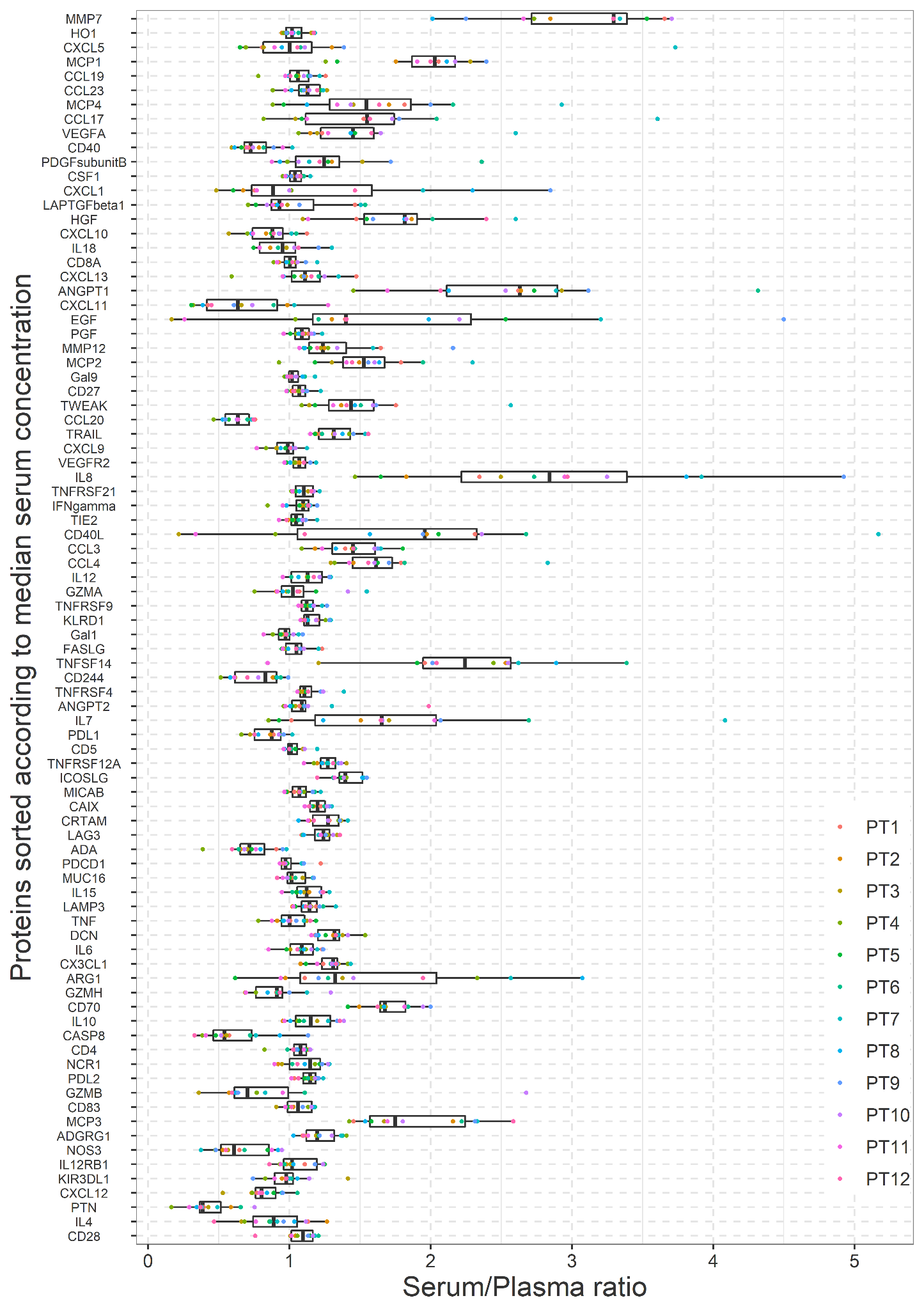


**Supplemental Figure 3:** **Correlation diagram and Passing-Bablok regression of serum vs. plasma value for all proteins.**

All plots show serum plotted against plasma. Each point represents a sample. Blue solid line = Passing-Bablok regression line. Blue dashed line = 95 % confidence interval of the regression. Red dashed line = identity line (x = y). Spearman correlation coefficient (r) are shown on each plot.

**
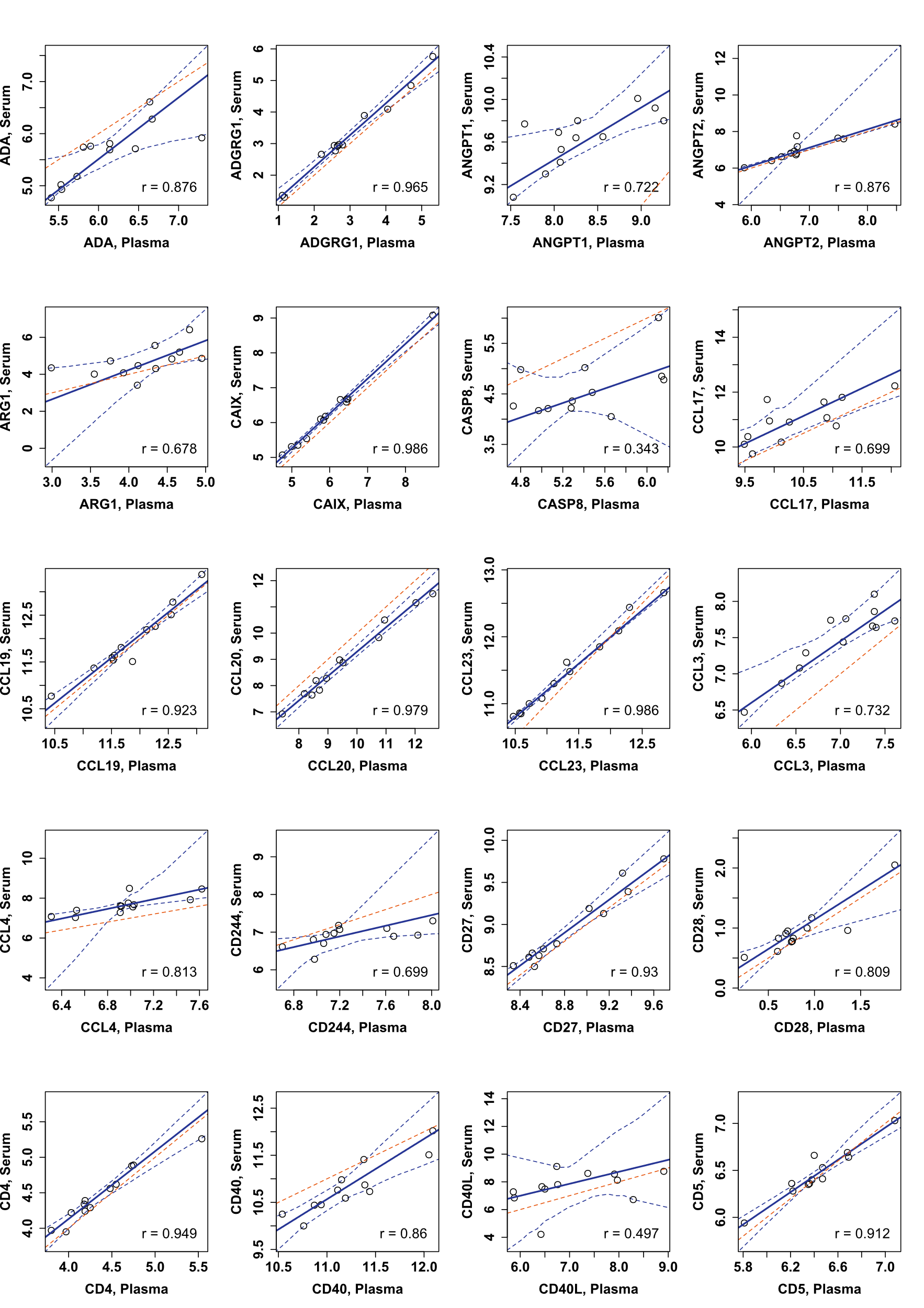
**

**
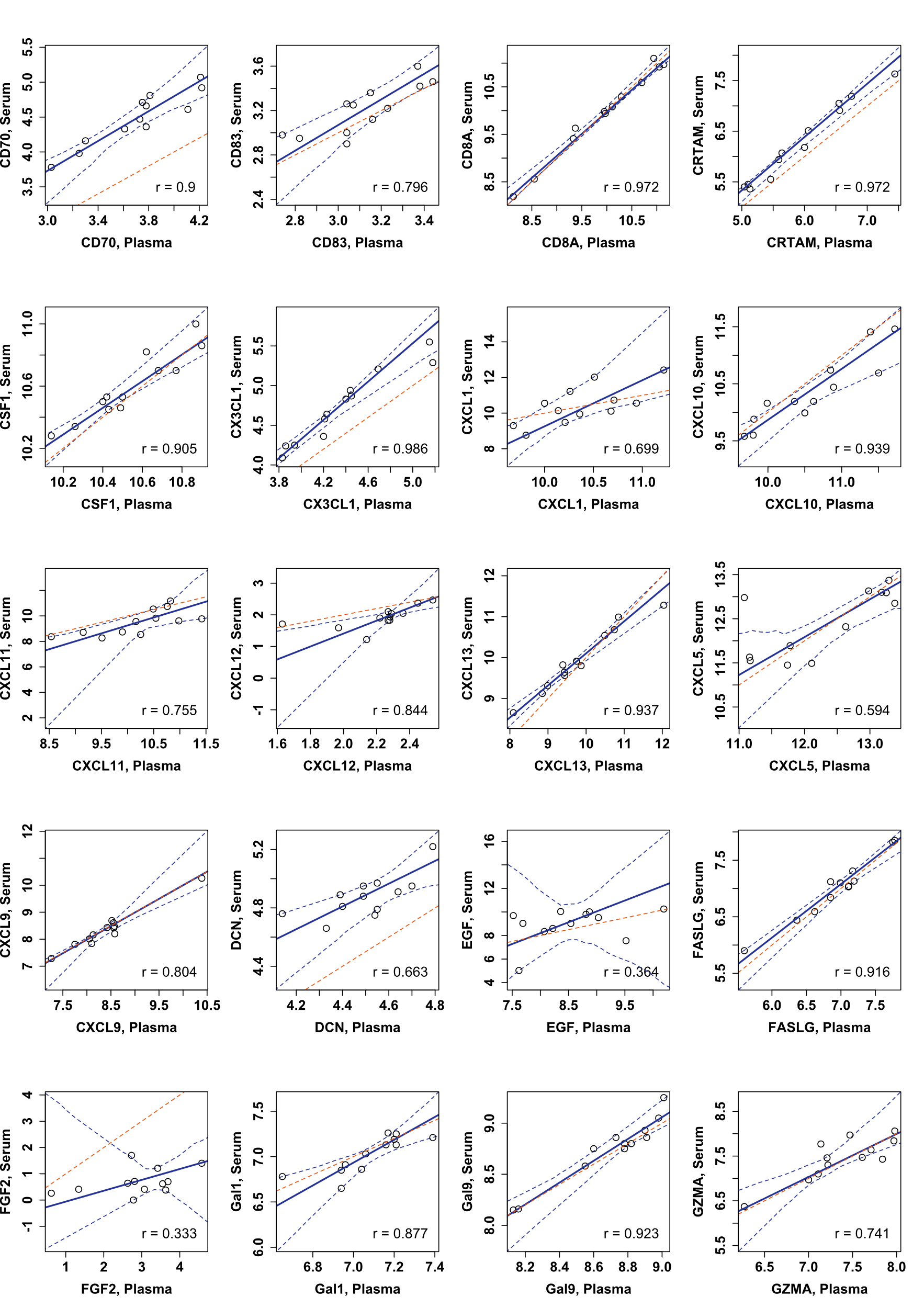
**

**
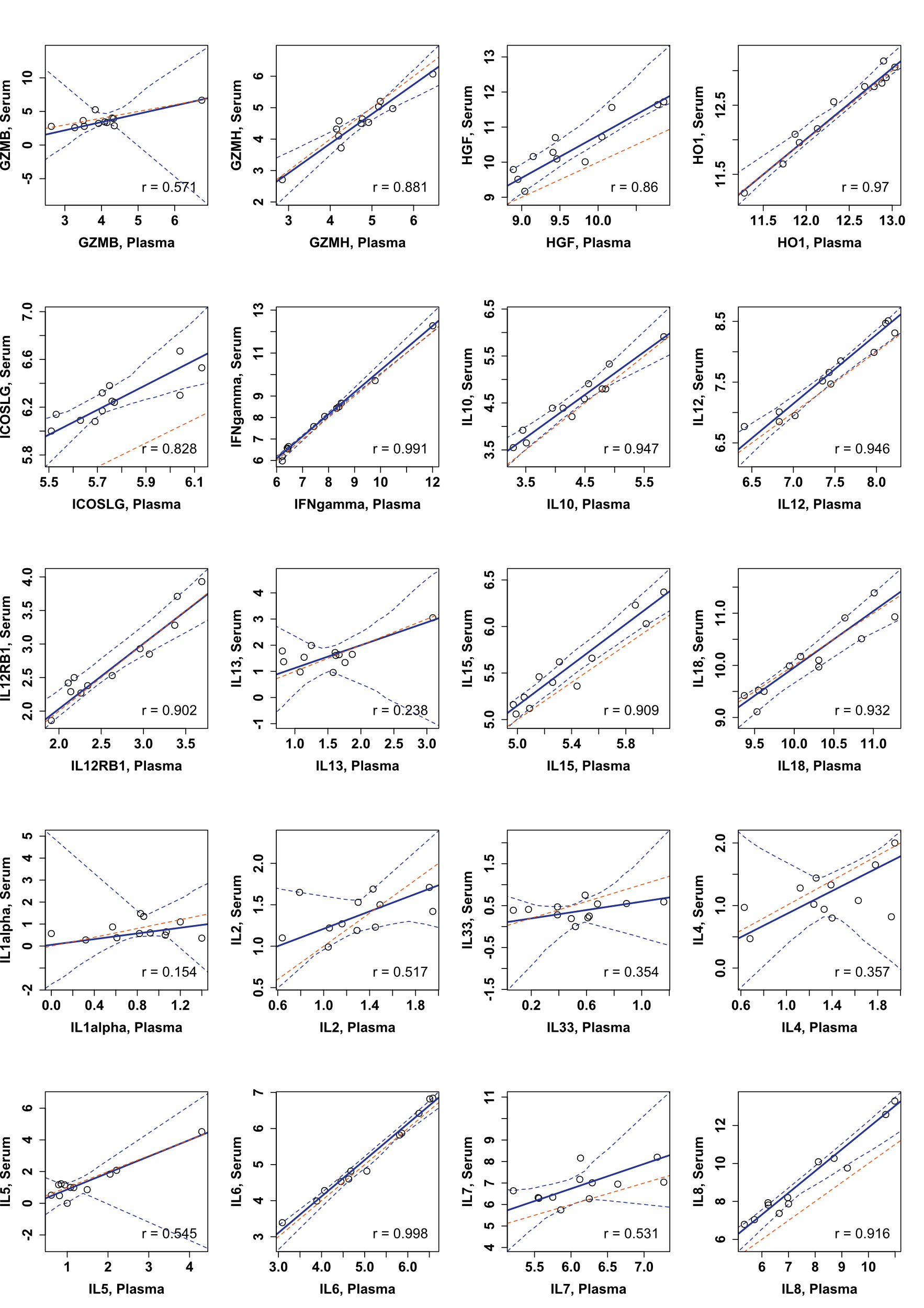
**

**
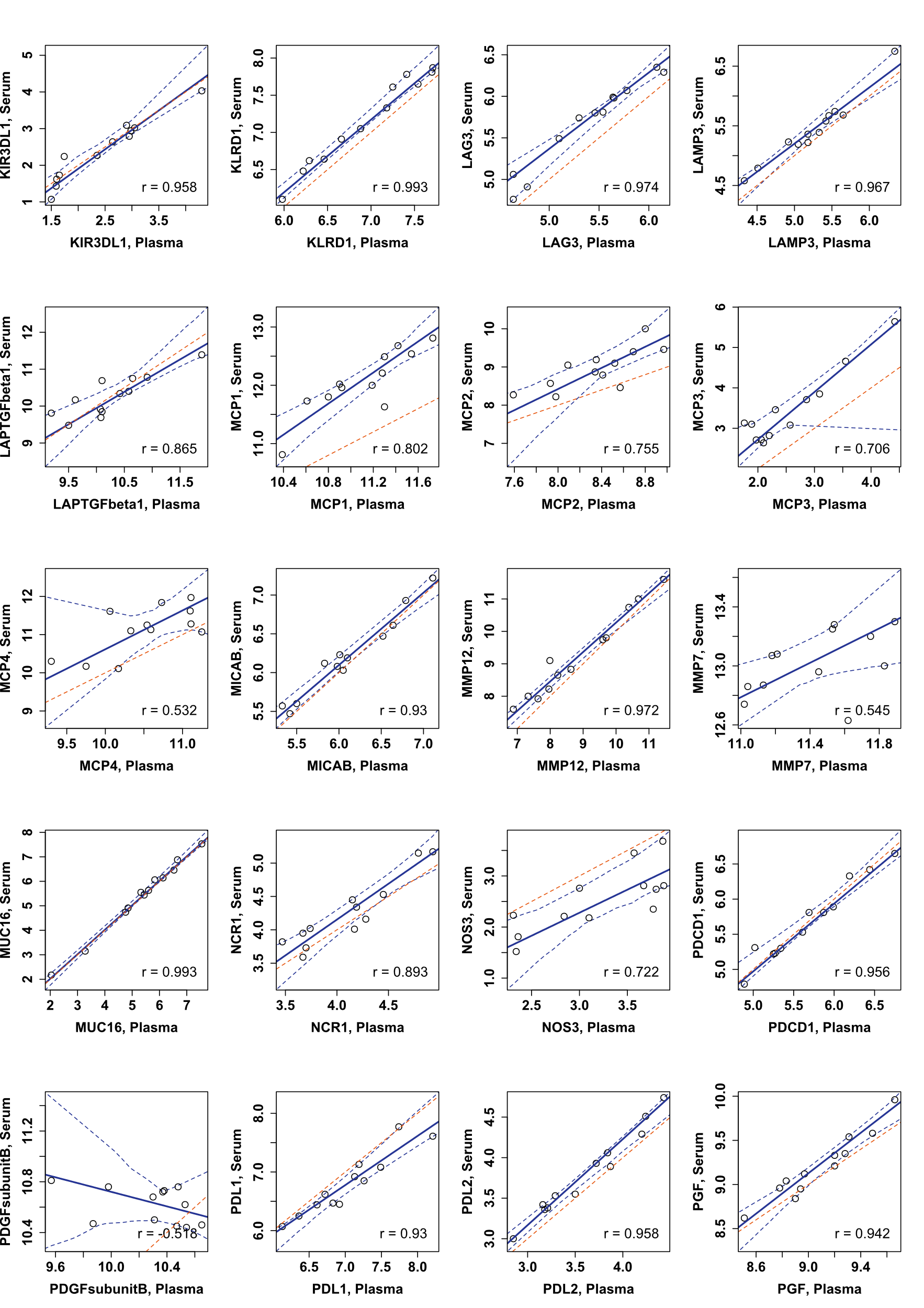
**

**
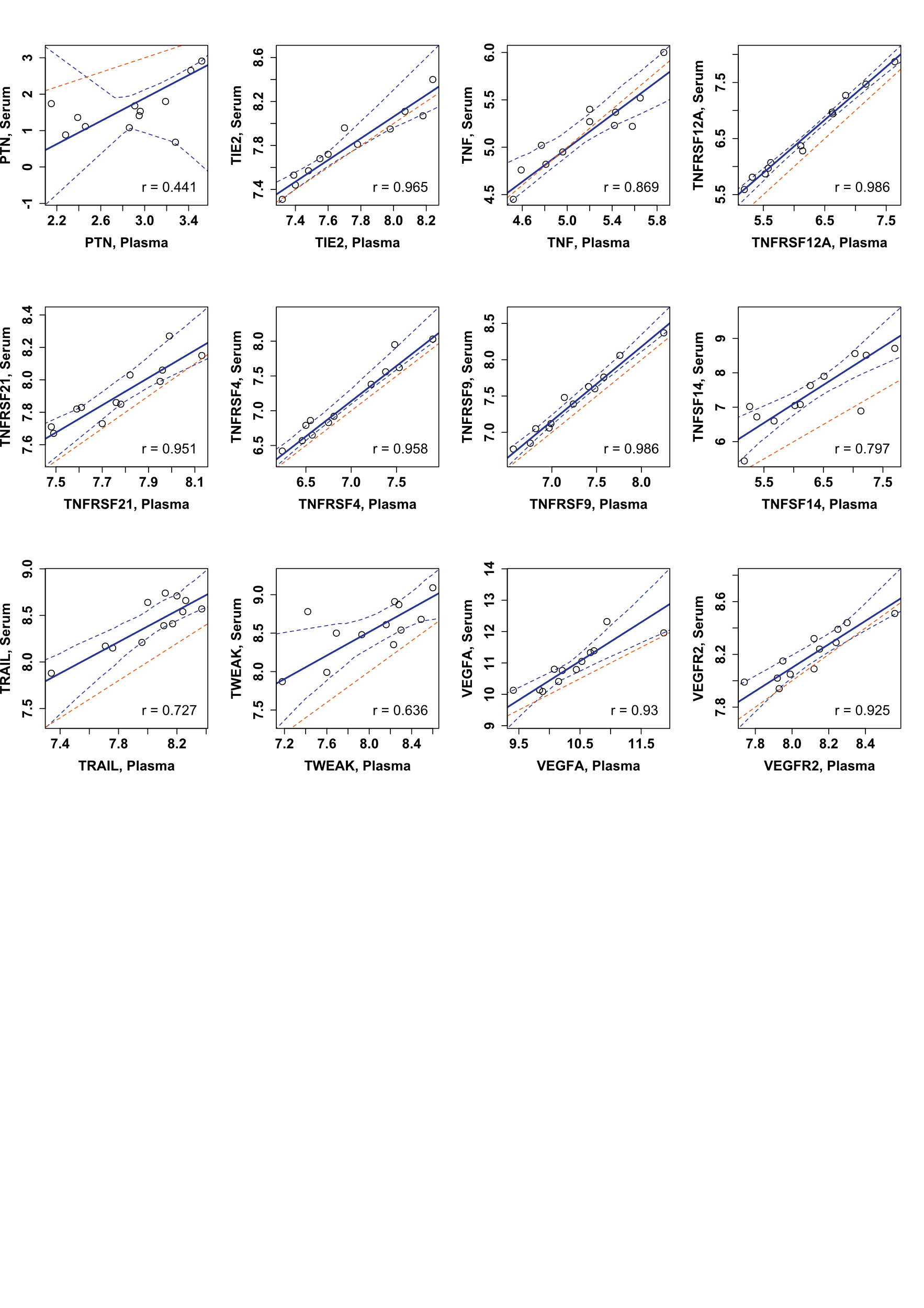
**

**Supplemental Figure 4**: **Bland-Altman plots of serum-to-plasma ratios**

Bland–Altman plots including the 5 proteins (ANGPT1, CXCL1, CXCL13, CCL23, and PDGF subunit B) with a significant association between ratio and protein concentration, and 4 proteins without an association. Dark solid line = mean ratio. Blue dashed line = 1 SD. Red dashed line = 2 SD. Green dashed line = linear regression line with 95% confidence interval highlighted.


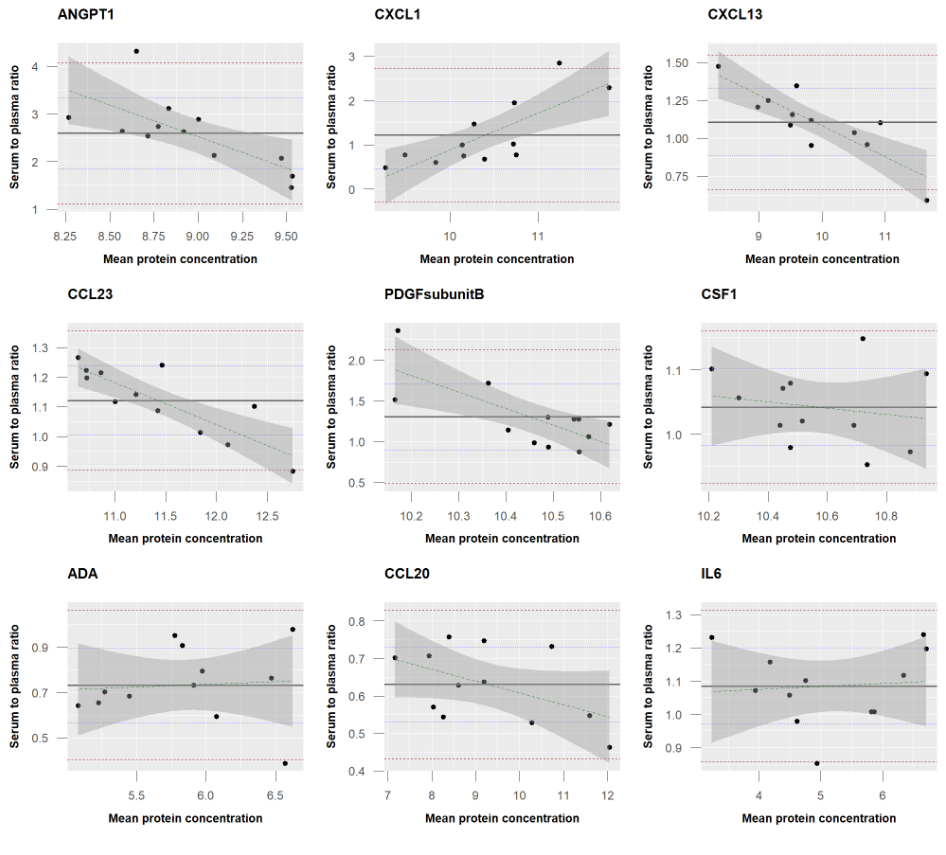


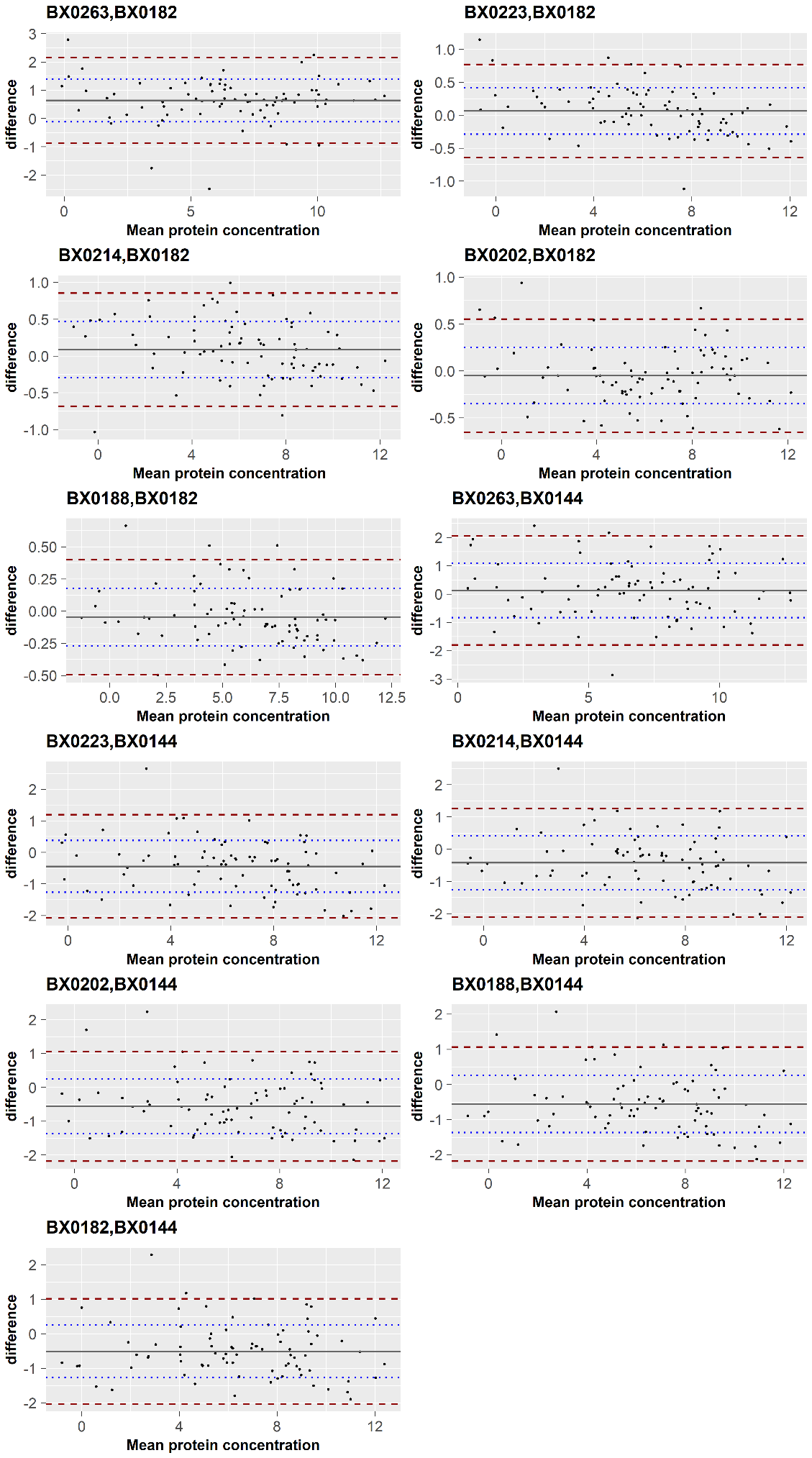
**Supplemental Figure 5**: **Bland–Altman plot comparing BX0144 with all other studies, and BX0182 with BX0188, BX0202, BX0214, BX0223, and BX0263.**

Mean concentration is plottet on x axis and the difference in median concentration between the studies is plotted on the y axis. The dark solid line = mean ratio. Blue dashed line = 1 SD. Red dashed line = 2 SD.
